# Antioxidant potential of tree bark extracts: Insight from the multi-level output of the Antioxidant Power 1 assay

**DOI:** 10.1371/journal.pone.0328790

**Published:** 2025-07-28

**Authors:** Johanna Rehrl, Thomas Sepperer, Sissy Häsler Gunnarsdottir, Thomas Schnabel, Gertie Janneke Oostingh, Anja Schuster

**Affiliations:** 1 Department of Health Sciences, Salzburg University of Applied Sciences, Salzburg, Austria; 2 Department of Biosciences and Medical Biology, Paris-Lodron University of Salzburg, Salzburg, Austria; 3 Department of Design and Green Engineering, Salzburg University of Applied Sciences, Salzburg, Austria; Universidad San Francisco de Quito - Campus Cumbaya: Universidad San Francisco de Quito, ECUADOR

## Abstract

Tree bark is a complex protective tissue that serves both physiological and defensive functions and is particularly rich in phenolic compounds bearing antioxidant, antimicrobial, anti-inflammatory and wound healing properties. The aim of this study was to investigate the antioxidant activity of aqueous bark extracts from 6 European tree species, namely black alder, common beech, silver birch, bird cherry, oak and scots pine using the antioxidant assay Antioxidant Power 1 (AOP1) on a keratinocyte cell line in the light of dermatological applications. The AOP1 assay relies on light-induced intracellular reactive oxygen species (ROS) production that disrupts efflux transport, enabling the accumulation of fluorescent cyanine dyes which can be quantitatively detected by increased fluorescence. Particular attention was placed on the multi-level output provided by AOP1, which includes information on the intracellular antioxidant as well as prooxidative effects of specific compounds and insight into the ground stress level of cells. The results showed that tree bark extracts exhibit a different antioxidant mechanism compared to the well-known antioxidative substance resveratrol. Bark extracts limit the total amount of ROS produced over the duration of the assay, with oak, beech and pine bark extracts showing the highest antioxidant capacity. In contrast, resveratrol delays ROS production over several illumination cycles before levels reach those of untreated cells. Cellular ground stress level was elevated by alder and birch whereas oak, beech and pine reduced the ground stress level similar to that of resveratrol. Results of AOP1 were linked to the constituents of the tree bark extracts derived by Soxhlet extraction, determined by HPLC-DAD analysis. The results highlight the potential of AOP1 as a screening tool with multi-level output and demonstrate the antioxidative potential of six European tree bark extracts, underscoring their promise as sustainable, value-added resources for the development of dermatological therapies targeting oxidative stress–related skin disorders.

## Introduction

The utilization of bioactive compounds derived from trees in different fields including medicine, food technology and cosmetics represents a common and rising research field [1,2]. Since trees are constantly subjected to a multitude of stressors, including environmental influences and pathogenic or herbivorous attacks, they have developed sophisticated mechanisms to protect themselves [3]. Phenolic compounds including flavonoids, phenolic acids, stilbenes, lignans or tannins are not only particularly fundamental to plant defense but also renowned for their antioxidant, antimicrobial, antiinflammatory and anticancerous effects [1,3–5]. These compounds possess antioxidant properties by acting as reducing agents, donating hydrogen molecules, quenching single oxygen molecules, chelating metals and activating antioxidant enzymes [6,7]. As an example, the well-known antioxidant resveratrol, chemically belonging to the stilbenes, is predominantly found in red grapes and wine. In addition to its antioxidant properties, resveratrol exhibits a range of therapeutic effects, including antiinflammatory, chemopreventive and cardioprotective effects. [8,9] Moreover, extracts derived from the bark of pine trees, particularly the *Pinus pinaster* species commercially available under the name Pycnogenol®, demonstrated strong antioxidant effects and a beneficial impact in the treatment of chronic diseases associated with inflammation and oxidative stress [10,11].

Furthermore, several dermatological conditions, including impaired wound healing, atopic dermatitis, *Acne vulgaris* and skin cancer might benefit from the use of natural antioxidants in therapy, due to the association of oxidative stress in these diseases [2,12].

The growing interest in the application of natural compounds as antioxidants has led to the development of numerous *in vitro* assays for the screening of potential therapeutic agents. Chemical *in vitro* assays based on hydrogen atom transfer, such as oxygen radical absorbance capacity measurement, and electron transfer, for instance 2,2-diphenyl-1-picrylhydrazyl (DPPH), are widely applied, but limited by the lack of molecular targets. Consequently, claims regarding antioxidative potential in target mechanisms must be critically examined. In addition, cell-based antioxidant assays are frequently employed due to their cost-effectiveness and feasibility compared to *in vivo* assays [13]. A widely used method is the dichlorofluorescein (DCF) assay, in which the properties of the chemical stressor determine the type of ROS induced. Stressors that trigger the formation of membrane-based lipid peroxyl radicals are often used, implying that only the antioxidant capacity against plasma membrane-based lipid peroxidation is assessed [14–17]. Additional constraints associated with these assays include the potential for unspecific oxidation of DCF [15,18] or the inability to differentiate between cytotoxic and antioxidative effects [16].

**Fig 1:**
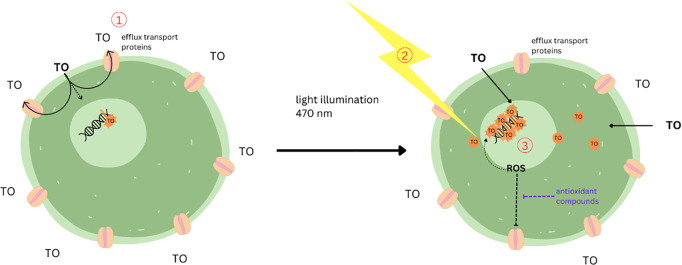
Mechanistic principle of the AOP1 assay to assess antioxidant potential. (1) healthy cells are able to efflux TO by drug efflux transport proteins leading to low fluorescence levels in this stage. (2) Light illumination (470 nm, 7.2 mJ/cm^2^) causes the formation of ROS, (3) resulting in a loss of cellular homeostasis affecting cellular functions such as the efficacy of drug efflux transport proteins which enables TO to target nucleic acids and increase fluorescence. [16] Created with Canva.com.

In comparison, the Antioxidant Power 1 (AOP1) assay, another *in vitro* assay to evaluate antioxidative capacity originally published by Gironde et al. [16], is able to differentiate antioxidative and prooxidative or cytotoxic effects. Its principle is based on the Light-Up Cell System (LUCS) approach to assess cell homeostasis proposed by Derick et al. [19]. The two principal components in both the LUCS and AOP1 assay, are the utilization of a biosensor, namely the cyanine dye thiazole orange (TO), and controlled cell illumination, which results in the formation of ROS. The AOP1 assay principle is illustrated in Fig 1 and can be described in three steps: (1) When cells are exposed to TO, TO targets nucleic acids, but – in their healthy state – cells are able to efflux TO utilizing drug efflux transport proteins. Due to the limitation of TO to its targets, the fluorescence detected in this stage is low. (2) In the second step, when cells are illuminated with blue light, the formation of ROS, including hydroxyl radicals and singlet oxygen molecules, is induced due to an energy transfer from TO to dioxygen. (3) The formation of ROS causes a loss of cellular homeostasis, which in turn affects cellular functions such as the efficacy of drug efflux transport proteins. This enables TO to remain in the cells and target nucleic acids resulting in a huge increase in fluorescence values. Potential antioxidants might limit the formation of ROS, protecting cellular homeostasis and cellular functions and thereby delaying or reducing the fluorescence increase which can be detected by the AOP1 assay. In the case of cytotoxic or prooxidant substances, which influence cell homeostasis, the fluorescence signal is already elevated prior to illumination, which is a key principle within the LUCS assay [16,19].

Within this study we utilized the LUCS approach to assess cell homeostasis and potential cytotoxic effects by determining the endpoint baseline fluorescence prior to any illumination as well as the principle of the above described AOP1 assay to asses dual prooxidant and antioxidant effects [16,19].

Utilizing the multi-level outputs of this assay we investigate the antioxidant potential as well as possible cytotoxic and prooxidant effects of aqueous bark extracts derived from 6 European tree species, namely black alder, common beech, silver birch, bird cherry, oak and scots pine. To investigate the potential of these extracts in relation to dermatological applications, a human keratinocyte cell line was used. To ascertain the bark extracts’ bioactive compounds, a quantitative analysis was conducted.This research seeks to provide insight into the sustainable valorization of tree bark as a bioactive resource and the need for advanced, cell-based screening tools such as the AOP1 assay to evaluate antioxidant efficacy.

## Materials and methods

### Bark extract preparation

Bark extracts were obtained from black alder (*Alnus glutinosa*), common beech (*Fagus sylvatica*), silver birch (*Betula pendula*), bird cherry (*Prunus padus*), oak (*Quercus spp*) and scots pine (*Pinus sylvestris*). For simplicity reasons, where applicable, the terms “alder”, “beech”, “birch”, “bird cherry”, “oak” and “pine” were used to refer to the bark extracts derived from these species. Bark was sampled from sawmills located in the region of Salzburg with the exception of bird cherry which was sampled in lower Austria. Prior to the extraction process, the crude bark samples (mixture of periderm, phloem and cambium) were dried at 60 °C for one week, grinded and filtered to a maximum size of 500 µm using a crossbeater mill (Retsch SK 100) and analytical screen machine (Retsch AS 200). For extraction, the Soxhlet extraction method was employed using 5 g biomass with 250 ml water as solvent during 18 h extraction time, followed by solid content and extraction yield determination. Prior to use in cell culture, the bark extracts were sterile filtered through filters comprising a polyvinylidene fluoride (PVDF) membrane and a pore size of 0.45 µm (Merck, Darmstadt, Germany).

### Total phenolic content

The total phenolic content (TPC) was determined colorimetrically using the Folin-Ciocâlteu method, which involves the reaction of phenols with phosphomolybdate and phosphotungstate. Accordingly, the protocol described by Ozturk et al. [20] was modified as follows:

200 µl of the extract (1 mg/ml) was diluted with 3 ml reverse osmosis water and subsequently 0.5 ml of Folin-Ciocâlteu reagent (Carl Roth, Karlsruhe, Germany) was added. This solution was vortexed three times for 10 s. After 3 min, 2 ml of 20% (w/v) sodium carbonate (VWR, Darmstadt, Germany) solution was added and vortexed again three times for 10 s each. Following 1 h of incubation in the dark, absorbance was measured at 765 nm using a VWR P4 UV/Vis spectrophotometer (VWR, Darmstadt, Germany). A calibration curve was constructed using gallic acid (Carl Roth, Karlsruhe, Germany) as the reference standard with an R^2^ value of 0.9997. The results are expressed as µg gallic acid equivalents (µgGAE)/mg dry extract.

### DPPH reduction assay

The antioxidative activity of the bark extracts was determined with the DPPH• radical reduction assay. The assay was performed according to Brand-Williams et al. [21] with modifications as follows:

The extracts were serially diluted from 2 to 0.063 mg/ml. 20 µl of each concentration was added to 2 ml of 6x10^-5^ M DPPH• (Sigma Aldrich, St. Louis, MO, USA) in methanol, and the solution was incubated for 1 h in the dark. Afterwards, the absorbance was measured at 515 nm with a VWR P4 UV/Vis spectrophotometer. The antioxidative activity was calculated using the following equation:


$$\;\%\;Inhibition=\;\frac{\left(A_{0}-A_{c}\right)}{A_{0}}\times 100$$ (1)

where *A*_*0*_ represents the DPPH• solution without extract and *A*_*c*_ denotes the absorbance of the different concentrations of the extracts or standard. Ascorbic acid (Carl Roth, Karlsruhe, Germany) was used as reference antioxidant. The quantity of extract required to achieve a 50% reduction in the initial DPPH• concentration was determined by constructing a scatter plot graph with regression between the concentration (x) and the percentage of inhibition (y). Automatic non-linear fit (Boltzmann) was applied to calculate the half maximal inhibitory concentration (IC_50_) using OriginPro 2023b. The results were expressed as IC_50_ values in dry extract per ml (µg/ml).

### HPLC-DAD

For the chemical characterization, high performance liquid chromatography coupled with diode array detection (HPLC-DAD) was conducted using the following configuration: a degassing unit DGU-20A, a liquid chromatograph LC-20AT, a column oven CTO-10AS with a C18 5 µm column 250 x 4.6 mm I. D. and a photodiode array detector SPD-M20A (all Shimadzu, Kyoto, Japan). The mobile phase was a linear gradient elution consisting of solvent A (water acidified with 0.1% trifluoracetic acid) and solvent B (methanol), both of which were HPLC gradient grade (Carl Roth, Karlsruhe, Germany). Extracts were filtered through a 0.45 µm PTFE syringe filter (Carl Roth, Karlsruhe, Germany) prior to injection without further dilution. The flow rate was set at 1 ml/min and the gradient was adopted from the method described by Mišan et al. [22] as follows: initial 10% B; 0–10 min. 10–25% B; 10–20 min. 25–60% B; 20–30 min. 60–70% B; 30–45 min. 70% B; 45–50 min. 70–10% B; 50–60 min. 10% B.

To facilitate identification and quantification, calibration curves (using the same parameters as described for extract analysis) were created for a range of standards, which included: Catechin (C), Epigallocatechin-Gallate (EC-G), dihydroxy benzoic acids (2,3; 2,4; 2,5; 2,6; 3,4; 3,5 DHBA), 4-hydroxybenzoic acid (4-HBA), vanillin (V), vanillic acid (VA), *para*-coumaric acid (*p*CA), *trans*-cinnamic acid (*t*CA), *trans*-3-hydroxy-cinnamic acid (*t*3-HCA), quercetin (Q), gallic acid (GA), ferulic acid (FA), syringic acid (SA) and ellagic acid (EA) (all VWR, Darmstadt, Germany or Carl Roth, Karlsruhe, Germany). Identification was conducted based on the analysis of the UV/Vis profile and/or the retention time [23]. For derivatives of compounds with no standards available, the quantification and identification was based on the UV profile and the calibration curve of representatives of the same group as described by Tsao et al. [24]. For instance, the concentration of unknown flavan-3-ols was calculated using the standard curve for catechin. Table 1 represents the data for UV/Vis wavelength (WL) used for detection, the coefficient for determination (R²) for the calibration curves and the limit of detection (LOD). Furthermore, the compounds were classified into subgroups, which are also listed in Table 1. The quantification of these subgroups was conducted by aggregating the quantities of the individual compounds within each subgroup. The software used for the HPLC analysis was LabSolutions Lite 5.97 (Shimadzu, Kyoto, Japan).

**Table 1:** Characteristics of the standards used for identification/quantification of the compounds present in the bark extracts including subgroup allocation.

Compound	UV/Vis detection WL [nm]	R²	LOD	Subgroup	Subgroup abbreviation
*GA*	271	0.9998	0.02	hydroxybenzoic acid	*HBA*
*3,4 DHBA*	258	0.9997	0.01	hydroxybenzoic acid	*HBA*
*3,5 DHBA*	307	0.9996	0.03	Hydroxybenzoic acid	*HBA*
*C*	278	0.9999	0.11	flavan-3-ols and glycosides	*F-3-Og*
*4 HBA*	258	0.9999	0.001	hydroxybenzoic acid	*HBA*
*2,5 DHBA*	330	0.9998	0.01	hydroxybenzoic acid	*HBA*
*VA*	258	0.9998	0.01	hydroxybenzoic acid	*HBA*
*SA*	271	0.9995	0.02	hydroxybenzoic acid	*HBA*
*V*	278	0.9999	0.01	aldehydes	*Ald*
*2,6 DHBA*	307	0.9997	0.02	hydroxybenzoic acid	*HBA*
*2,4 DHBA*	258	0.9999	0.01	hydroxybenzoic acid	*HBA*
*2,3 DHBA*	244	0.9998	0.02	hydroxybenzoic acid	*HBA*
*pCA*	310	0.9999	0.001	cinnamic acid derivatives	*CA-D*
*FA*	322	0.9999	0.01	cinnamic acid derivatives	*CA-D*
*t3-HCA*	310	0.9999	0.01	cinnamic acid derivatives	*CA-D*
*EA*	367	0.9998	0.08	ellagic acid	*EA*
*tCA*	273	0.9997	0.01	cinnamic acid derivatives	*CA-D*
*Q*	367	0.9998	0.02	flavonoids	*Fla*
*EC-G*	274	0.9997	0.03	flavan-3-ols and glycosides	*F-3-Og*

### Cell culture

The HaCaT cell line, spontaneously immortalized human keratinocytes, was purchased from Cell Lines Service in Germany and was cultured in Dulbecco’s Modified Eagle Medium (DMEM) high glucose (4.5 g/l) (Sigma-Aldrich, St. Louis, MO, USA). The medium was supplemented with 2 mM stable L-glutamine (Capricorn, Ebsdorfergrund, Germany), 1% Penicillin-Streptomycin (10,000 units penicillin and 10 mg streptomycin/ml) (Sigma-Aldrich, St. Louis, MO, USA) and 10% FBS (Capricorn, Ebsdorfergrund, Germany) and is referred to as growth medium. HaCaT cells were maintained at 37 °C in a humidified atmosphere with 5% CO_2_ and split once a week to varying cell densities in T75 cell culture flasks (Sarstedt, Nümbrecht, Germany). For splitting or seeding, the old medium was discarded, cells were washed with phosphate buffered saline (PBS; Gibco life technologies, Carlsbad, CA, USA) and detached utilizing TrypLE Express (Gibco life technologies, Carlsbad, CA, USA) according to the manufacturer’s instructions.

### Antioxidant power 1 (AOP1) assay

To determine the intracellular antioxidative effect of bark extracts and to gain insight into the cytotoxicity of bark extracts towards HaCaT cells, the AOP1 assay was performed.

In each experiment an untreated control (UT) and resveratrol (62.5 µM; Carl Roth, Karlsruhe, Germany) dissolved in ethanol (Merck, Darmstadt, Germany) as reference antioxidant were included. Dilutions of bark extracts and controls were prepared in DMEM without FBS and PhenolRed (Sigma-Aldrich, St. Louis, MO, USA), which is used for all experimental steps and referred to as medium. Since the ethanol content for the reference antioxidant was 0.625%, this ratio was maintained during the dilution process for all samples including the bark extract treatments and the untreated control.

A total of 3.25 x 10^4^ cells/ well were seeded in flat black clear bottom 96-well microtiter plates (Corning Incorporated, Corning, NY, USA) in 100 µl growth medium, with the edged wells left empty (100 µl PBS) and 3 wells reserved for the thiazole orange (TO)-only control. Following an incubation overnight at 37 °C and 5% CO_2_, the cells were treated with bark extracts (reaching concentrations between 50 and 800 µg/ml), resveratrol (final concentration 62.5 µM) or medium in case of the untreated control. After 3 h of incubation at 37 °C and 5% CO_2_, the treatment medium was discarded and 100 µl of 4 µM TO (AAT Bioquest, Pleasanton, CA, USA) diluted in medium was added in the dark and incubated for 1 h at 37 °C and 5% CO_2_. The first fluorescence detection was conducted prior to illumination with excitation and emission wavelengths of 495 and 535 nm a Tecan Spark Cyto plate reader (Tecan, Grödig, Austria) maintained at 37 °C to assess the ground stress levels of the cells. Afterwards a kinetic fluorescent detection comprising 30 cycles of illumination (96 LEDs, 470 nm, 7.2 mJ/cm^2^; Tecan, Grödig, Austria) and fluorescence detection with excitation and emission wavelengths of 495 and 535 nm was performed.

### AOP1 data normalization

In the context of data analysis, a normalization procedure was performed with the initially derived relative fluorescence units (RFU) of the AOP1 assay (see Eq (2), adapted from Gironde et al. [16] and Held et al. [25]). Following TO background subtraction, the number of light flashes at which the fluorescence profile of the untreated control reaches a plateau (*FN*_*plateau(UT)*_) was determined. The relative fluorescence units at each flash number (*RFU*_*FNx*_) were normalized to the plateau value of the untreated control (*RFU*_*plateau(UT)*_), subtracting the baseline fluorescence (*RFU*_*FN0*_ or *RFU*_*FN0(UT)*_). In case the baseline fluorescence *RFU*_*FN0*_ was elevated by more than 10% of the untreated control, these treatment conditions were excluded from further analysis due to cytotoxic or prooxidant effects.


$$NFU\;\;\%=\;\frac{{RFU}_{FNx}-\;{RFU}_{FN0}}{{RFU}_{FNplateau(UT)}-{RFU}_{FN0(UT)}}$$ (2)

S1 Fig depicts the process of normalization in oak treated cells.

To enable comparison between different antioxidants after normalization, the cellular antioxidant index (*CAI*) was calculated. Part of the calculation of the *CAI*, as performed by Gironde et al. [16], shown in Eq (3), involves integration, i.e., the calculation of the AUC, of the respective treatment condition curve ${AUC}_{x}=\;\int_{0}^{FNplateau(UT)}{NFU}_{FNx}$ and the untreated control curve ${AUC}_{UT}=\;\int_{0}^{FNplateau(UT)}{NFU}_{FN(UT)}$.


$$CAI=1000-1000*\;\frac{{AUC}_{x}}{{AUC}_{UT}}$$ (3)

### Statistical analysis

For TPC and DPPH three independent experiments were performed. HPLC-DAD was performed once. For the AOP1 assay three independent experiments were conducted in triplicates for each experimental condition. Raw data was normalized in Microsoft Excel Version 2308, while the area under the curve (AUC) was calculated using GraphPad Prism 8 software version 8.0.1. Statistical analysis was performed in GraphPad Prism 8 employing ordinary one-way ANOVA and Dunnett’s multiple comparison test with a single pooled variance. p values are indicated as ns (not significant) p > 0.05 (not displayed in graphs), * p ≤ 0.05, ** p ≤ 0.01, *** p ≤ 0.001 and **** p ≤ 0.0001. All relevant data points are provided in the Supplement.

## Results and discussion

### Determination of total phenolic content and chemical antioxidant activity of tree barks

Evaluation of the phytochemical properties via TPC revealed the highest TPC content in alder, followed by birch and oak (Table 2). These findings were in accordance with the results of the DPPH• assay, which also demonstrated the highest antioxidative potential in alder, followed by birch and oak. In contrast, the lowest TPC content was observed in bird cherry. In the DPPH• assay, ascorbic acid was used as the reference antioxidant, exhibiting an IC_50_ value of 230 µg/ml, which was comparable to that of the oak and birch bark extracts. Only alder showed higher antioxidative activity, with an IC_50_ value of 152 mg/ml. Even though a number of studies investigated the chemical composition of tree bark of the species of interest, a direct comparison of the TPC and antioxidant activity of the investigated tree species to available literature is limited due to differences in extraction methods and solvents applied [2,26–32]. Additionally, the polyphenol composition of bark is influenced by factors such as tree age, altitude, habitat, and the vertical position of sampling, which were not specifically controlled for in this study [33,34].

**Table 2:** Extraction yield of the tree bark material, total phenolic content (TPC) and DPPH• IC_50_ of the bark extracts.

Tree bark extract	Extraction yield [%]	TPC [µg GAE/mg extract] *		DPPH• IC_50_ [µg/ml]*
Alder	25.0	425	± 6.12	152 ± 2.9
Beech	7.2	173.3	± 2.97	831 ± 15
Birch	7.64	328.64	± 0.5	243 ± 63
Bird Cherry	11.72	153.8	± 2.34	402 ± 73
Oak	6.64	263.2	± 0.89	238 ± 20
Pine	8	189.2	± 2.27	476 ± 71

* mean ± = standard deviation (SD) of n=3.

### HPLC-DAD analysis

The compounds identified by HPLC-DAD were classified into 6 categories. Table 3 demonstrates the quantified results for the 6 barks, expressed as mg/100 g of dried bark. Birch exhibited the highest concentration of catechin and other flavan-3-ols with over 1100 mg/100 g dried bark, which aligns with the TPC determination, where birch shows the most pronounced response after alder. Various (hydroxy) cinnamic acid derivatives were found in the extracts of alder and bird cherry, with concentrations of 600 mg/100 g dry bark and 250 mg/100 g dry bark, respectively. These findings are consistent with the low IC_50_ concentrations for alder and the moderate IC_50_ concentrations for bird cherry. Oak was the only bark extract in which sufficient ellagic acid was present to be quantifiable (454 mg/100 g dry bark), which accounts for its relatively high TPC.

**Table 3:** Quantification of the compounds identified by HPLC-DAD for the 6 bark extracts.

Tree bark extract	*HBA*	*F-3-Og*	*CA-D*	*Fla*	*EA*	*Ald*
Alder	78.92	180.01	600.43	–	–	–
Beech	106.04	–	8.80	–	–	12.15
Birch	22.10	1132.00	–	–	–	–
Bird Cherry	57.84	21.08	249.20	–	–	–
Oak	67.73	25.27	–	3.70	454.32	–
Pine	54.92	25.60	16.08	6.32	–	23.52

Quantification and identification results within the 6 categories *HBA* (hydroxybenzoic acids), *F-3-Og* (flavan-3-ols and glycosides), *CA-D* (cinnamic acid derivatives), Fla (flavonoid), *EA* (ellagic acid) and *Ald* (aldehydes) of the 6 bark extracts derived from HPLC-DAD. Values expressed as mg/100g dry bark of n = 1.

**Fig 2:**
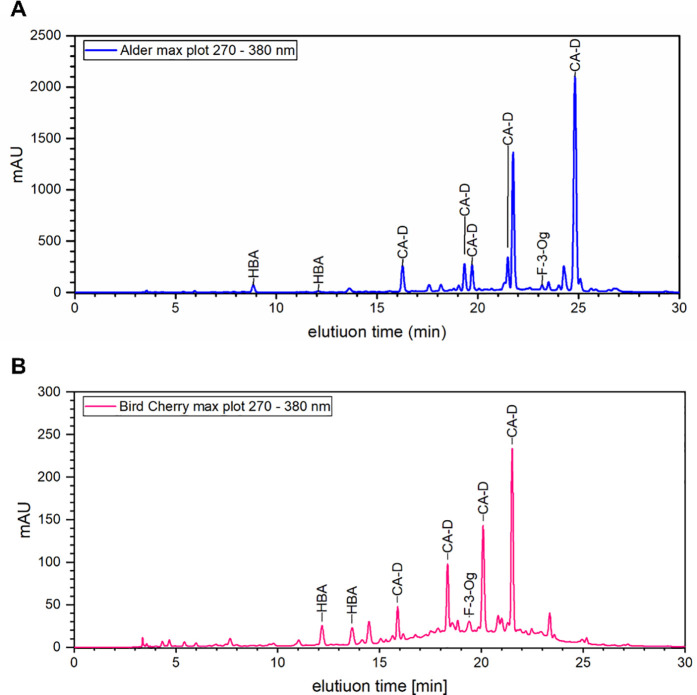
Chromatograms of alder and bird cherry. Exemplary chromatograms (max plot 270–380 nm) for alder (A) and bird cherry (B) extract showing the adsorption against the elution time. The compound categories were assigned to the corresponding peaks.

Additionally, Fig 2 demonstrates representatively the chromatograms for alder (A) and bird cherry (B), with the identified compounds of the 6 subgroups marked.

### Bark extracts limited ROS production differently from resveratrol

**Fig 3:**
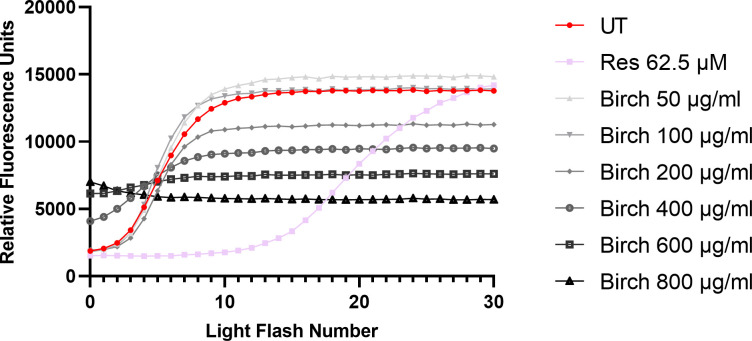
Fluorescence profiles of untreated cells, cells treated with different birch bark extract concentrations or resveratrol. Birch bark at 50 µg/ml reached a higher plateau level compared to UT. Resveratrol (Res, 62.5 µM) showed a delayed increase in fluorescence reaching the plateau at light flash number 30. In contrast high concentrations of birch bark extracts (400–800 µg/ml) resulted in increased baseline fluorescence indicating cytotoxic or prooxidant behavior. Data from one replicate (no standard deviation).

The AOP1 assay was performed to determine the antioxidant power of 6 different bark extracts. Resveratrol, a known antioxidant, was used as a positive control. Comparing the fluorescent profiles of bark extracts and the reference antioxidant (resveratrol) treated cells revealed a clear distinction. While resveratrol delayed the fluorescence increase for approximately 10 illumination cycles and gained a plateau near the untreated control, some bark extracts showed minor delay but a pronounced decrease in the fluorescence plateau level compared to the untreated control in a concentration-dependent manner (see S1 FigA Oak 100–800 µg/ml, Fig 3 Birch 200 µg/ml). This indicates that some bark extracts prevented excessive ROS formation over an extended period of illumination cycles. A possible explanatory mechanism could be a prolonged scavenging ability of compounds within the bark extracts resulting in TO binding to DNA and continued TO export by drug efflux transport proteins. This effect of limiting ROS to a certain degree over an extended period of time has been observed by Gironde et al. for some concentrations of other known antioxidants, such as α-tocopherol, epigallocatechin, quercetin, epicatechin and menadione tested on HepG2 cells. However, this effect was not further discussed. A comparison of the raw fluorescence profiles of these compounds with the bark extracts revealed that especially α-tocopherol at 250 µM resulted in a slight reduction in the fluorescence plateau, exhibiting a behavior similar to the bark extracts [16]. This effect requires further investigation, including the testing of other known antioxidant substances that may have a comparable effect. With further research, this mechanism might be of benefit to the cosmetics sector, especially for the attenuation of UV-induced ROS upon photodamage, or to the food industry, particularly in the context of preservation.

In contrast, other bark extracts resulted in increased baseline fluorescence indicating cytotoxic or prooxidant behavior (Fig 3 Birch 400–800 µg/ml) or a higher plateau level (Fig 3 Birch 50 µg/ml) in relation to the untreated control both indicating contribution to ROS formation. A contributing factor to the distinct mechanisms between the bark extracts and pure resveratrol may be that the crude bark extracts consist of several components at lower concentrations, which may act in an additive, synergistic or antagonistic manner [35,36]. However, the cause for the distinct mechanisms necessitates further investigation. Additionally, it is challenging to conduct comparative analyses due to the shortage of available literature on the AOP1 assay.

### Quantification of ground stress level through baseline fluorescence

**Fig 4:**
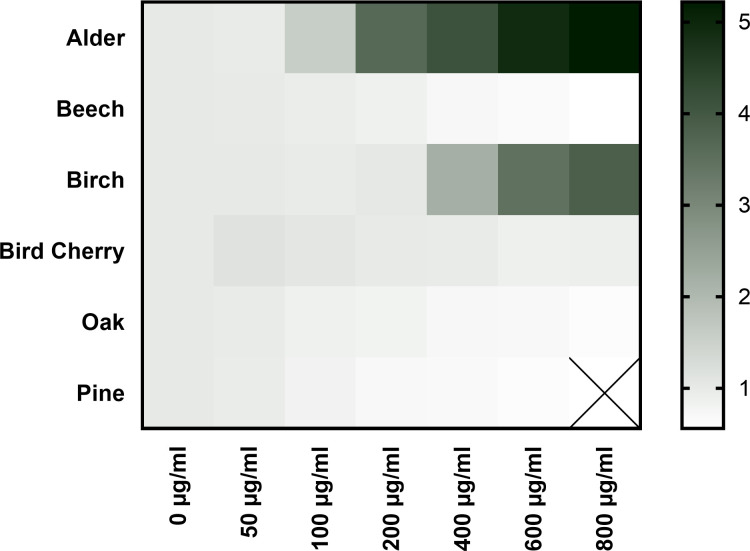
Conditional formatting of baseline fluorescence of bark extract treated HaCaT cells in relation to UT cells. UT cells were set to 1 (0 µg/ml). Dark colors represent cells with increased ground stress, indicating a prooxidative or cytotoxic effect, while lighter colors (white) represent concentrations of treatments that reduced ground stress. Values represent the mean (n = 3).

The baseline fluorescence detected after compound treatment but prior to the first illumination was set in relation to the untreated control (set to 1) and as mentioned above, extracts exceeding a fluorescence increase threshold of 10% of the untreated control were excluded from further analysis. Fig 4 represents the relation of the fluorescence base levels between the UT (0 µg/ml) and the treated cells in a heat map. Fluorescence ratios exceeding 1 are depicted with stronger color intensity (black), thereby signifying elevated fluorescence and prooxidative or cytotoxic effects. This effect may be explained by an imbalanced homeostasis due to ROS and thus by cell entry and DNA intercalation of TO prior to illumination. Conversely, lighter colors (white) correspond to concentrations exhibiting a basal fluorescence ratio below 1, which indicates a reduction in basal stress.

Most pronounced prooxidative effects were observed for alder, beginning at a concentration of 100 µg/ml showing a 5-fold increase at 800 µg/ml and birch from 400 µg/ml, reaching a nearly 4-fold increase at 800 µg/ml. In contrast, the dose-response relationship of bird cherry was inverse (i.e., the lower the concentration, the higher the baseline fluorescence). However, the fluorescence base level remained comparable to that of the UT control, apart from 50 µg/ml which exceeded the 10% threshold. Other bark extracts, especially beech, oak and pine, reduced baseline fluorescence in a concentration-dependent manner (by approximately 40% at the highest concentration tested), indicating lower ROS levels after extract treatment compared to the untreated control. In comparison, the baseline fluorescence of resveratrol (62.5 µM) treated cells was reduced by about 30% (not shown in heatmap).

In the literature, reduced baseline fluorescence is visible for SHSY5Y and Caco-2 cells after treatment with various concentrations of Aronia extract, but the authors did not address this effect [37]. The ability to detect baseline fluorescence is an advantage offered by AOP1 compared to other *in vitro* antioxidative assays, as it allows an insight into the initial state of the cells, in this case after 3 h of treatment, compared to the untreated control. Especially when screening a large number of compounds, this insight into potential prooxidative, cytotoxic or cytoprotective effects of the treatment compounds could significantly reduce the time required for cytotoxicity and/or viability tests. To ascertain the consistency of the observations, a comprehensive study would be necessary, in which the AOP1 baseline fluorescence results are compared with those from cytotoxicity and viability assays.

### Evaluation of the CAI values reveals distinct antioxidant capacity

**Fig 5:**
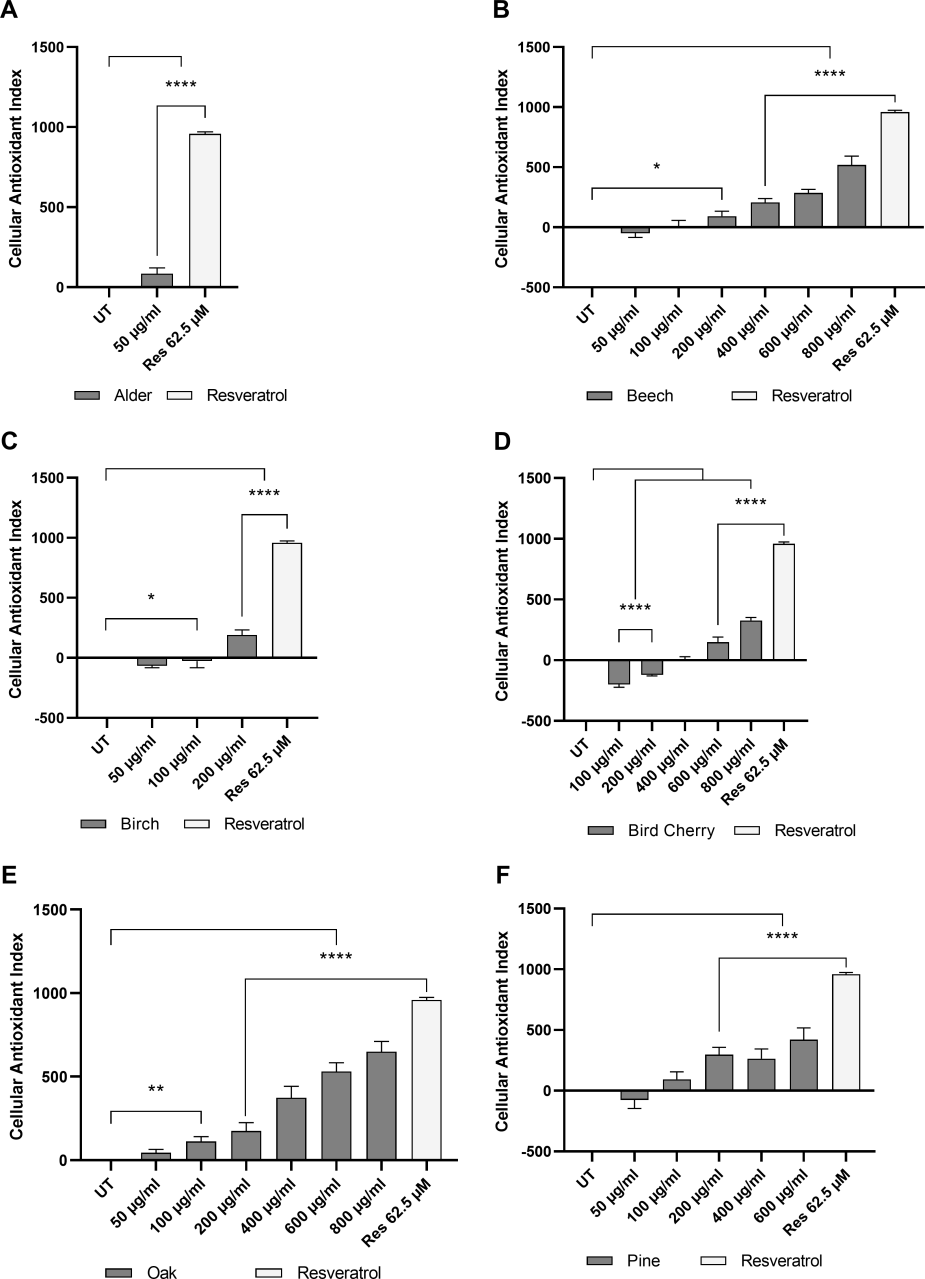
Cellular antioxidant index values. Average CAI values of (A) alder, (B) beech, (C) birch, (D) bird cherry, (E) oak and (F) pine bark extract (50 to 800 µg/ml) treated cells. n = 3, error bars represent SD, statistical significance: * p ≤ 0.05, ** p ≤ 0.01, *** p ≤ 0.001 and **** p ≤ 0.0001.

Gironde et al. [16] suggest the generation of dose-response profiles using the CAI values and non-linear regression to calculate efficacy concentrations leading to half maximum response (EC50), where feasible. Given the distinctive mechanism of action of bark extracts, only partial or no effect was evident, precluding the calculation of EC50 values. However, the *CAI* values were calculated for all bark extract concentrations and resveratrol (62.5 µM; *CAI* = 959) and are summarized in Table 4 and Fig 5. The CAI values for low concentrations of beech, birch, bird cherry and pine exhibited negative values, deriving from fluorescence profiles with a faster increase and/or a higher plateau level than the UT control. During integration this resulted in a higher AUC than untreated control and finally in negative values for the CAI using Eq (3). In addition, similar effects were observed with Quercetin (lowest concentration tested, 7.8 µM) and γ-Tocopherol (various concentrations) by Gironde et al. [16]. These compound concentrations indicate prooxidative behavior; however, further investigations are required to verify their prooxidant effect.

**Table 4:** Average *CAI* values of bark extract treated cells (0 (UT) to 800 µg/ml).

Species	0 µg/ml (UT)	50 µg/ml	100 µg/ml	200 µg/ml	400 µg/ml	600 µg/ml	800 µg/ml
Alder	0	84 (****)	PO	PO	PO	PO	PO
Beech	0	−49	9	93 (*)	207 (****)	285 (****)	520 (****)
Birch	0	−67 (*)	−27	192 (****)	PO	PO	PO
Bird cherry	0	PO	−199 (****)	−121 (****)	2	150 (****)	327 (****)
Oak	0	45	111 (**)	174 (****)	374 (****)	530 (****)	649 (****)
Pine	0	−76	94	298 (****)	264 (****)	421 (****)	x

“PO” (pro-oxidative) indicates concentrations exhibiting elevated baseline fluorescence. Negative values (gray) also indicate prooxidative behavior. *CAI* values represent the mean of three replicates (n = 3) with the associated significance levels provided in brackets. Statistical significance: * p ≤ 0.05, ** p ≤ 0.01, *** p ≤ 0.001 and **** p ≤ 0.0001.

Alder and birch acted prooxidant in concentrations higher than 50 or 200 µg/ml respectively, however alder at 50 µg/ml and birch at 200 µg/ml demonstrated significant antioxidative potential (alder *CAI* = 84, birch *CAI* = 192). Bird cherry exhibited prooxidative behavior at concentrations of up to 200 µg/ml, while at higher concentrations (600–800 µg/ml) moderate antioxidative effects could be observed (S2 Fig). Oak and beech exhibited the most pronounced antioxidative effect between 50 or 100 and 800 µg/ml, reaching maximum *CAI* values of 649 and 520, respectively. In comparison to the other bark extracts, pine did not show a conventional dose-response relationship, as evidenced by the absence of a linear correlation between concentration and *CAI* values. Instead, it exhibited a higher *CAI* at 200 µg/ml than at 400 µg/ml, reaching a maximum at 600 µg/ml.

In a study assessing the antioxidant potential of 15 known antioxidants towards HepG2 cells with the AOP1 assay, Gironde et al. also identified antioxidants that exhibited only a partial effect (astaxanthin) or no effect (α- and γ-tocopherol, vitamin E acetate and sulforaphane). Consequently, no EC50 could be calculated for these antioxidants. Another study by Dufour et al. revealed a *CAI* value of approximately 900 when treating Caco-2 cells with Aronia extract at a concentration of 625 µg/ml. The concentration range for calculating the *CAI* and the EC50 was between 0 and 2500 µg/ml in a two-fold serial dilution with an EC50 at 349.1 µg/ml [37]. This may be an indication that at concentrations exceeding the stock concentration of the extracts examined in this study, the CAI would be higher and an EC50 could be calculable. However the comparison should be interpreted with caution due to differences in the normalization procedure [16,25].

Cinnamic acid derivatives, mainly present in alder (600.43 mg/100 g dried bark) and to a lesser extent in bird cherry (249.20 mg/100 g dried bark), possess many beneficial properties including antioxidative (both direct and indirect), antiinflammatory, antimicrobial and antitumor effects [38,39]. Cinnamic acid is a key metabolite of the shikimate pathway and a precursor of many different phenolic compounds such as flavonoids, coumarin or lignin in the general phenylpropanoid pathway [3,40,41]. Furthermore, the presence of hydroxybenzoic acids and flavan-3-ols and their glycosides was detected in both extracts, with a higher concentration observed in alder. Supported by the highest TPC and lowest IC_50_ (DPPH) value of alder, this suggests that alder should have a strong antioxidant effect. However, at a concentration of 100 µg/ml and above it was strongly prooxidative. Gryko et al. reported evidence that some highly concentrated phenolic compounds might promote oxidation by Fe(III)-Fe(II) reduction leading to hydroxyl radical formation, which at some concentrations might contribute to the prooxidative behavior of alder or other extracts with similar properties [41].

In contrast, birch has an extremely high content of Falvan-3-ols and glycosides (1132.0 mg/100 g dried bark), which is consistent with the high TPC and low IC_50_ (DPPH) values, indicating a strong antioxidant effect. At 200 µg/ml, birch possess one of the highest *CAI* values compared to the other extracts, however at higher concentrations prooxidant or cytotoxic properties were observable which might be due to antagonistic effects of the single compounds.

The data generated by the AOP1 assay suggest that oak has the greatest antioxidant potential of all extracts examined, which is also confirmed by the low IC_50_ (DPPH) and high TPC values. The most abundant compound in oak was ellagic acid, which is best known for its antioxidant capacity, in addition to its antiinflammatory, chemopreventive, cardioprotective, antidiabetic and neuroprotective effects. Ellagic acid acts as metal chelator, reduces lipid peroxidation and induces antioxidant enzymes including catalase or glutathione peroxidase as well as non-enzymatic antioxidants such as glutathione [42].

## Conclusion

This study demonstrates the potent capacity of six European tree bark extracts to inhibit intracellular ROS, with oak bark showing the highest efficacy. Using the AOP1 assay, a sensitive, cell-based method capable of detecting both antioxidative and prooxidative effects in real time, the extracts were evaluated for their impact on baseline and induced oxidative stress levels in living cells. These findings not only underscore the value of the AOP1 assay as a versatile screening tool but also highlight the therapeutic potential of wood bark, an underutilized by-product of the timber industry, as a sustainable source of bioactive compounds for dermatological applications.

## Supporting information

S1 Fig AOP1 fluorescence profiles before and after normalization. Fluorescence profiles of resveratrol (Res, 62.5 µM) and oak bark extract treated cells (A) before normalization, where the untreated control reached a plateau after 13 cycles (FNplateau(UT)) and (B) after normalization towards the plateau value of the control.
(TIF)

S2 Fig Raw fluorescence profiles of alder, beech and pine bark extract treated HaCaT cells derived by the AOP1 assay. Raw fluorescence profiles of alder (A and B), beech (C) and pine (D) treated cells. Data from one replicate (no SD).
(TIF)

S1 File Supplement and data repository. (ZIP)
