# Supplementary figures and images for: Antioxidant potential of tree bark extracts: Insight from the multi-level output of the Antioxidant Power 1 assay
Source: PLoS One. 2025 Jul 28;20(7):e0328790. doi: 10.1371/journal.pone.0328790 (PMC12303309; doi:10.1371/journal.pone.0328790)

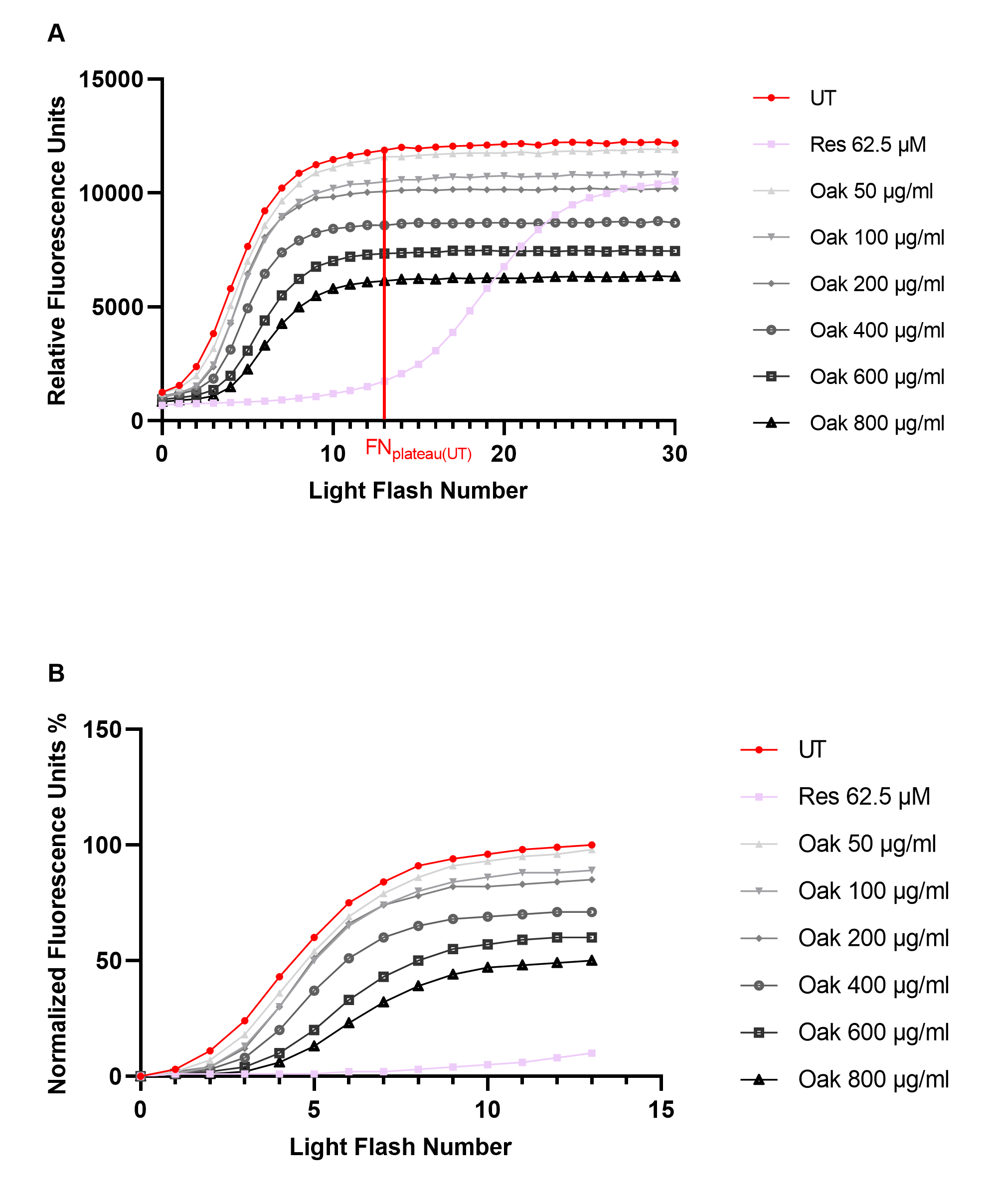

Supplement: S1 Fig — Fluorescence profiles of resveratrol (Res, 62.5 µM) and oak bark extract treated cells (A) before normalization, where the untreated control reached a plateau after 13 cycles (FNplateau(UT)) and (B) after normalization towards the plateau value of the control. (TIF) [file pone.0328790.s001.tif]

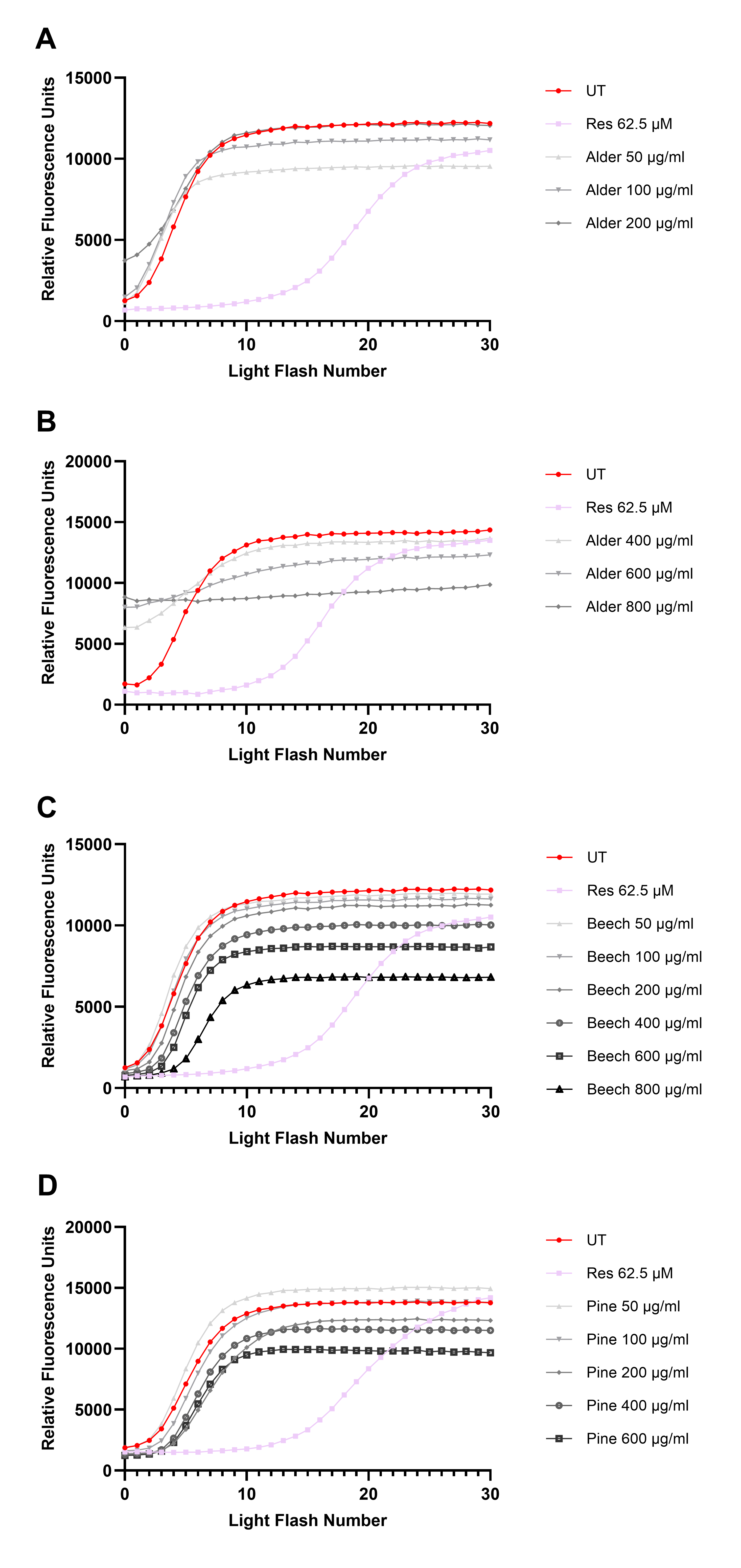

Supplement: S2 Fig — Raw fluorescence profiles of alder (A and B), beech (C) and pine (D) treated cells. Data from one replicate (no SD). (TIF) [file pone.0328790.s002.tif]
