# Supplementary material for: Antioxidant potential of tree bark extracts: Insight from the multi-level output of the Antioxidant Power 1 assay
Source: PLoS One. 2025 Jul 28;20(7):e0328790. doi: 10.1371/journal.pone.0328790 (PMC12303309; doi:10.1371/journal.pone.0328790)
Supplement: S1 File — (ZIP) [file pone.0328790.s003.zip › Rehrletal_Supplement_PlosOne_revised.docx]

*Supplement*

**Antioxidant Potential of Tree Bark Extracts: Insights from the Multi-Level Output of the Antioxidant Power 1 Assay**

Johanna Rehrl ^a,b^, Thomas Sepperer ^c^, Sissy Häsler Gunnarsdottir ^a,c^, Thomas Schnabel ^c^, Gertie Janneke Oostingh ^a^, Anja Schuster ^a*^

^a^ Department of Health Sciences, Salzburg University of Applied Sciences, Salzburg, 5412, Austria

^b^ Department of Biosciences and Medical Biology, Paris-Lodron University of Salzburg, Hellbrunner Straße 34, Salzburg, 5020, Austria

^c^ Department of Design and Green Engineering, Salzburg University of Applied Sciences, Salzburg, 5412, Austria

* corresponding author: anja.schuster@fh-salzburg.ac.at

**S1 Fig: AOP1 fluorescence profiles before and after normalization.** Fluorescence profiles of resveratrol (Res, 62.5 µM) and oak bark extract treated cells (A) before normalization, where the untreated control reached a plateau after 13 cycles (*FN_plateau(UT)_*) and (B) after normalization towards the plateau value of the control.

**S2 Fig. Raw fluorescence profiles of alder, beech and pine bark extract treated HaCaT cells derived by the AOP1 assay.** Raw fluorescence profiles of alder (A and B), beech (C) and pine (D) treated cells. Data from one replicate (no SD)


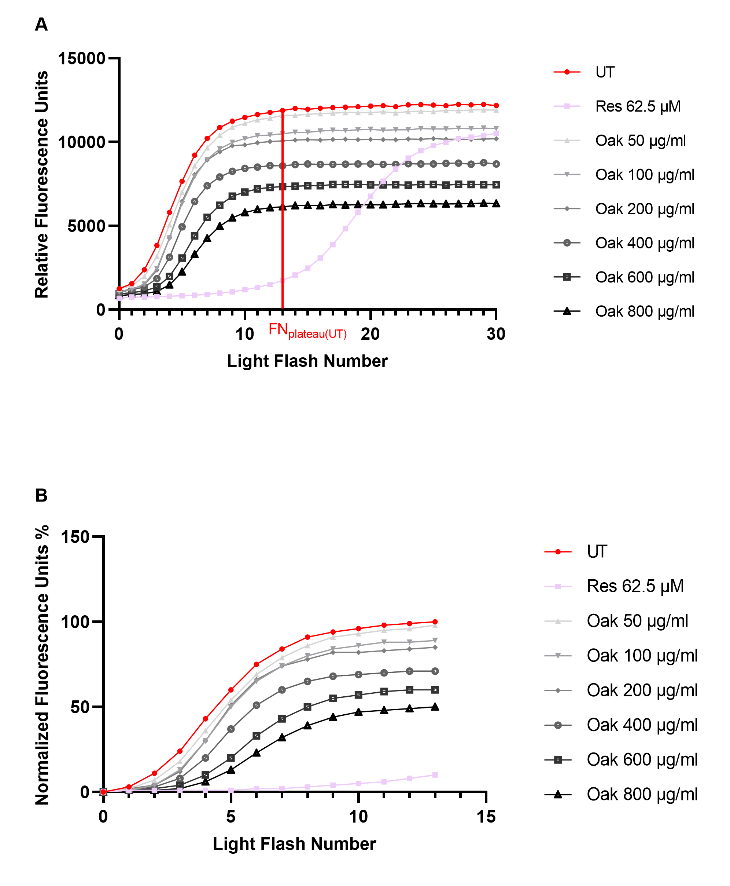


**S1 Fig: AOP1 fluorescence profiles before and after normalization.** Fluorescence profiles of resveratrol (Res, 62.5 µM) and oak bark extract treated cells (A) before normalization, where the untreated control reached a plateau after 13 cycles (*FN_plateau(UT)_*) and (B) after normalization towards the plateau value of the control.


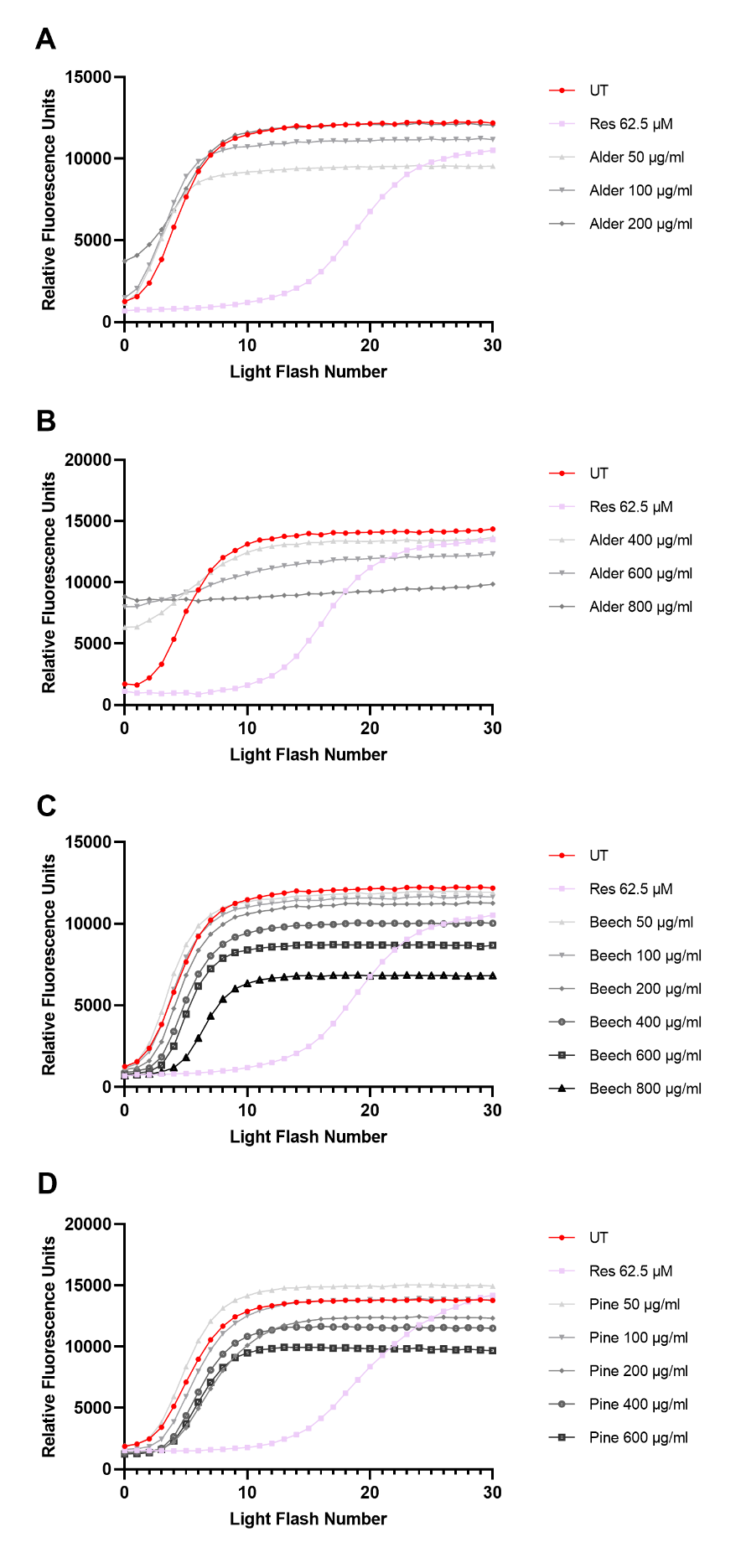


**S2 Fig. Raw fluorescence profiles of alder, beech and pine bark extract treated HaCaT cells derived by the AOP1 assay.** Raw fluorescence profiles of alder (A and B), beech (C) and pine (D) treated cells. Data from one replicate (no SD)
